# Artificial Intelligence and Multiple Sclerosis: Up-to-Date Review

**DOI:** 10.7759/cureus.45412

**Published:** 2023-09-17

**Authors:** Yahya Naji, Mohamed Mahdaoui, Raymond Klevor, Najib Kissani

**Affiliations:** 1 Neurology Department, REGNE Research Laboratory, Faculty of Medicine and Pharmacy, Ibn Zohr University, Agadir, MAR; 2 Neurology Department, Agadir University Hospital, Agadir, MAR; 3 Neurology Department, University Hospital Mohammed VI, Marrakech, MAR; 4 Neuroscience Research Laboratory, Faculty of Medicine and Pharmacy, Cadi Ayyad University, Marrakech, MAR

**Keywords:** neuroimaging, prognosis, diagnosis, multiple sclerosis, artificial intelligence

## Abstract

Multiple sclerosis (MS) remains a challenging neurological disorder for the clinician in terms of diagnosis and management. The growing integration of AI-based algorithms in healthcare offers a golden opportunity for clinicians and patients with MS. AI models are based on statistical analyses of large quantities of data from patients including “demographics, genetics, clinical and radiological presentation.” These approaches are promising in the quest for greater diagnostic accuracy, tailored management plans, and better prognostication of disease. The use of AI in multiple sclerosis represents a paradigm shift in disease management. With ongoing advancements in AI technologies and the increasing availability of large-scale datasets, the potential for further innovation is immense. As AI continues to evolve, its integration into clinical practice will play a vital role in improving diagnostics, optimizing treatment strategies, and enhancing patient outcomes for MS.

This review is about conducting a literature review to identify relevant studies on AI applications in MS. Only peer-reviewed studies published in the last four years have been selected. Data related to AI techniques, advancements, and implications are extracted. Through data analysis, key themes and tendencies are identified. The review presents a cohesive synthesis of the current state of AI and MS, highlighting potential implications and new advancements.

## Introduction and background

Artificial intelligence (AI) in healthcare entails the development and deployment of intelligent systems and algorithms that can learn from data, identify patterns, and perform tasks usually requiring human intelligence [1]. These algorithms process and analyze diverse healthcare data, including electronic health records (EHRs), medical images, genomic data, and clinical research papers, to generate insights and support clinical decision-making [1,2]. By analyzing medical images and clinical data, AI algorithms (AIA) can assist healthcare professionals in making accurate diagnoses. Studies have demonstrated the potential for AI systems to achieve comparable or even superior diagnostic accuracy to human experts in various medical fields [2].

AI could also suggest personalized treatment plans by integrating patient data, clinical guidelines, and research evidence. Through the analysis of patient-specific factors like genetic profiles, medical history, and treatment responses, AIA can optimize treatment selection and dosage. This optimization enhances treatment efficacy while minimizing adverse effects [3]. AI can identify potential drug targets, predict drug efficacy, and design novel molecules, leading to the development of new therapeutic interventions for conditions such as cancer, Alzheimer's disease, and multiple sclerosis [4]. In terms of monitoring systems, AI enables continuous tracking of patients' health status, vital signs, and disease progression outside of conventional healthcare settings. This technology allows early detection of changes in patient conditions, facilitates remote consultations, and enhances patient engagement in self-care, thus leading to improved outcomes and reduced healthcare costs [2,3,5].

Multiple sclerosis is a chronic autoimmune disease affecting the central nervous system, characterized by the inflammation and damage of myelin sheaths surrounding nerves along with neurodegeneration. The disease affects more than 2.8 million people worldwide, this equates to one in 3,000 people living with multiple sclerosis (MS) [6]. It is known to cause various debilitating symptoms such as ataxia, sensory impairment, cognitive dysfunction, and fatigue [7]. MS has different phenotypes which are relapsing-remitting MS (RRMS), clinical isolated syndrome (CIS), and progressive MS (PrMS) [7]. The diagnosis of MS is delicate. Both clinical and MRI findings could be found in other central nervous system (CNS) inflammatory diseases. Moreover, precise diagnosis of MS is imperative to control disability and progression of the disease [8]. AI has therefore become a tool in increasing demand in MS management, especially in the field of neuroradiology. AI lends itself well to predicting clinical disability and the long-term benefits and safety of disease-modifying treatments (DMT) in patients with MS [9]. In this review, we focus on the recent advancements and implications of AI techniques that are used for the diagnosis, monitoring, and treatment of MS.

## Review

Literature review

A literature search about AI and MS was conducted in August 2023 for peer-reviewed journal articles published in PubMed between January 2018 and December 2022. The study was based on the Preferred Reporting Items for Systematic Reviews and Meta-Analysis (PRISMA) statement guidelines. Articles were collected using the following keyword search: “artificial intelligence” (AND) “multiple sclerosis,” ({Artificial intelligence (MeSH Terms)} AND {Multiple sclerosis (MeSH Terms)} AND {2018/01/01:2022/12/31(dp)} AND {english (la)}). Articles were judged to be relevant upon meeting the following inclusion criteria: articles must be an indexed Medline study, published in the last four years (2018-2022) and examine the advancements and implications of AI in MS, articles must not be about proceedings, and conference abstracts and papers not published in English.

**Figure 1:**
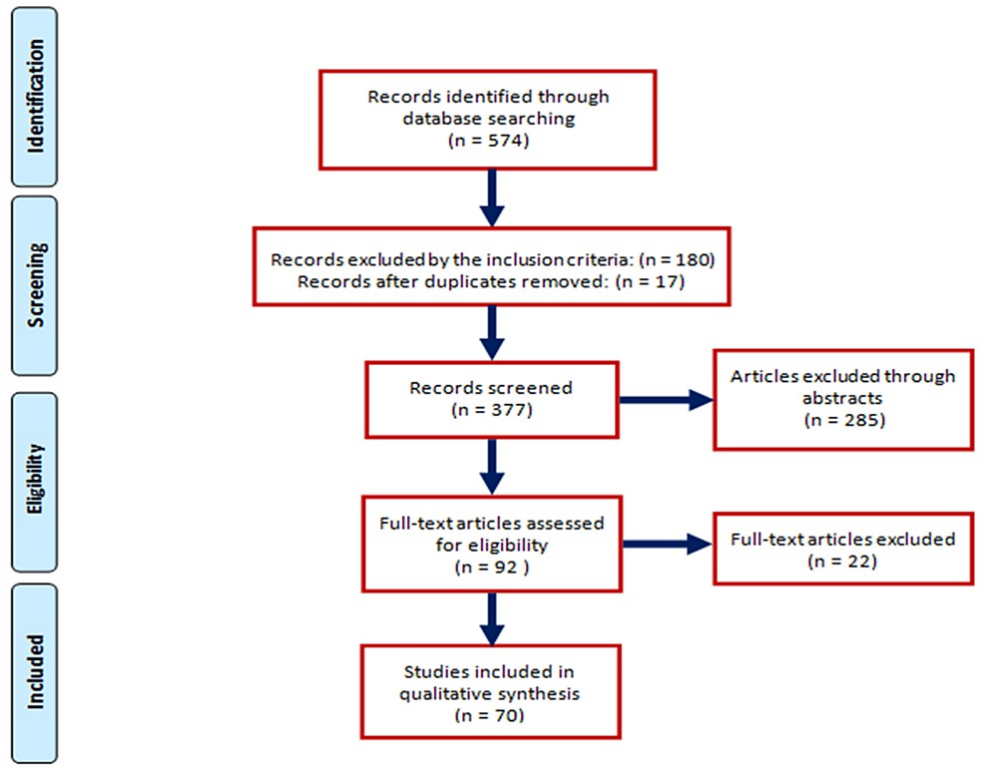
PRISMA flow diagram depicting the flow of information through the different phases of a systematic review. PRISMA: Preferred Reporting Items for Systematic Reviews and Meta-Analysis

The PubMed database search yielded 574 articles (1989-2022). The majority of the studies treating AI and MS were published in the last four years “394 articles (2018-2022) vs. 180 articles (1989-2017).” After eliminating duplicate materials and resources incompatible with our inclusion criteria, 385 articles remained, and through critical analysis of study abstracts and full-text articles, 70 articles were included in our study (Figure 1).

Full-text review of the 70 papers led to the identification of three key themes which were categorized into the following: (1) diagnosis and imaging analysis of MS disease (40 papers). (2) Disease progression and prognosis prediction of MS disease (23 papers). (3) Treatment selection and optimization of MS disease (seven papers).

Diagnosis and imaging analysis of MS disease

AI has shown remarkable potential in aiding the diagnosis of MS by analyzing medical imaging data, such as magnetic resonance imaging (MRI) scans [9,10]. Studies have demonstrated the efficacy of AI in automating lesion segmentation and quantification, which facilitates accurate diagnosis of MS [11,12]. This is particularly beneficial as it reduces the manual effort and time required by radiologists and neurologists to analyze the images [13]. AI algorithms can accurately detect and outline new active lesions, enabling faster and more consistent identification [14-16]. It can also assist in quantifying the burden of MS lesions (by analyzing the size, location, and number of lesions), to aid in assessing disease severity [17,18].

AIA are capable of recognizing patterns and abnormalities within medical images that may be indicative of MS and could identify subtle changes in brain and spinal cord structures associated with MS lesions [19,20]. The implementation of computer-aided diagnosis system (CADS) and deep learning (DL) help distinguish MS-related abnormalities from other conditions and contribute to accurate diagnosis [21-23]. For precise diagnosis of MS, several automated or semi-automated methods use lesional imaging biomarkers, such as cortical lesions (CL), the central vein sign (CVS), and paramagnetic rim lesions (PRL) [24]. Others use a supervised classification approach by using an adaptive dictionary learning method for automated classification of multiple sclerosis lesions in MRI [25]. Furthermore, the 3D MRI fingerprinting and functional/diffusion MRI modalities have the ability to differentiate between MS and healthy controls with a high rate of accuracy (89%±2%) [26,27].

Recent studies show the efficacy of automatic delineation models in lesion segmentation and reproduction of a quantitative analysis of lesion loads [28-30]. They even recommend their implementation in clinical routine [31-34]. Some studies report the role of computer-aided diagnosis (CAD) methods by magnetic resonance spectroscopy (MRS) in distinguishing between multiple sclerosis lesions and low-grade brain tumors [18,35]. Others use cortical lesion AI-based assessment to support clinical decisions at 7T MRI, or artificial neural network using a convex combination of infinite kernels, especially in the field of differential diagnosis [17,36,37]. Specific AI models predict MS phenotype and disability, through machine learning models based on MRI, Expanded Disability Statue Scale (EDSS) scores, and biomarker data [19,38,39]. Many papers report the use of convolutional neural network models (CNNs) and Bayesian network approach [40,41].

Another field of AI in MS diagnosis is optical coherence tomography (OCT), which is usually used to detect optic neuritis in MS patients. Through time, MS patients show progressive thinning of retinal layers, accelerated by relapses and disease progression [42]. A recent review describes the adaptation of OSCAR-IB criteria, used for OCT quality control, to incorporate AI [43,44]. Several machine learning models (MLMs) distinguish successfully MS patients from healthy controls (e.g., support vector machines {SVMs} and decision trees) [45]. Moreover, two recent studies evaluated prognostic markers in MS using MLMs on small sample sizes, they got ambitious results with automated image pattern recognition [42,44,45].

New implications of AI in MS concern biological biomarkers. A recent study described that a support vector machine with 10-fold validation allows predicting MS with high sensitivity, specificity, and accuracy of 92.9%, due to a combination of four antioxidants biomarkers (zinc, adiponectin, total radical-trapping antioxidant parameter {TRAP} and sulfhydryl groups {SH}) [46]. Another study succeeds with a machine learning workflow to build MS stage-specific classifiers based on peripheral blood mononuclear cells (PBMCs). The PBMCs contain specific dysregulations in genes and pathways, which help classify the MS stage (CIS, RRMS, and PrMS) [47]. Other modalities of AI are being employed in monitoring balance disorders, tremor diagnosis, and automated analysis of visual evoked potentials [9,48,49].

Disease progression and prognosis prediction of MS disease

The integration of clinical data in the assessment of MS using AI has become usual in the last few years [50]. For example, the ProMiSi project predicts the disease's progression and prognosis based on general demographic information and EDSS score, with a high rate of accuracy [51]. In addition, the integration of gait data and MLMs can offer a practical patient-centric approach to aid clinicians in monitoring MS [52]. The use of an instrumented treadmill or a walkway helps detect gait disturbances by extrapolating the patient results to standard gait variables, which definitely helps to monitor the disease progression [52,53].

AI facilitates long-term monitoring of MS, by the digitization of all patient clinical data in order to track disease activity [54]. The miniaturization of sensors and computational progress allow the gathering of digital patient outcomes and electronic health records (EHR) [54]. Many researchers have developed a wearable lifestyle sensor based on conceptual models (such as Sensor, Observation, Sample, and Actuator {SOSA} and the Web Annotation Data Model {WADM}) in conjunction with telemedicine and online apps to facilitate clinical supervision and decision making [54,55]. Also, MLMs were used to automatically analyze voice recordings of MS patients and detect slight changes, the results seem promising for MS diagnosis and progression tracking [56].

Cognitive impairment is common in MS patients, so accurate measures of cognition could be used as markers of disease progression [57]. Some authors proposed a test named the “Integrated Cognitive Assessment (ICA),” which is self-administered and language-independent [58]. They have compared its correlation, sensitivity, and accuracy with the Brief International Cognitive Assessment for MS (BICAMS) [58]. The results showed that the ICA could be used as a digital marker of cognitive impairment, especially for its high accuracy rate (AUC=95%) [58].

Others have digitalized the Brief Visuospatial Memory Test-Revised (BVMT-R) using a convolutional neural network (CNN) to rate the drawings of MS patients. As a result, the accuracy of the BVMT-R in identifying optical recognition deficits and their progression was about 80% [59]. In a recent study, a combination of the Symbol Digit Modalities Test (SDMT) performance and MRI neuroimaging (global tissue volume, global diffusion tensor imaging) was also performed [60]. The authors conclude that bilateral nucleus accumbens and right thalamus volume loss go along with a worse SDMT score. The high accuracy of this model seems to be a potential detector of subtle cognitive changes [60].

AI and large-scale data sharing could easily predict the progression of MS, which is crucial for treatment planning and optimizing patient outcomes [61,62]. It covers a large field from MRI image analysis “T2-fluid-attenuated inversion recovery (FLAIR), 2D T1-weighted images (WI), 3D T1-WI, diffusion tensor imaging, and functional magnetic resonance imaging” to large multidomain datasets [61,63].

MRI is used for tracking disease progression through different modalities. Several studies used convolutional neural networks (CNNs) for assessment of volumetric lesion load and lesion activity segmentation in time [64-66]. Others combined advanced MLMs and sensitive techniques, such as diffusion tensor imaging to estimate MS patients' disability along with regional grey matter volumes and functional connectivity [67-69]. Some papers confirm that thalamic volume loss was a significant predictor of disability and cognitive decline, they have used deep learning approach based on Deep Gray Rating via AI [70-72].

Treatment selection and optimization of MS disease

AI algorithms have a crucial task in guiding treatment selection and optimization for MS patients. In order to achieve personalized care in MS, authors have proposed a three-step roadmap as follows: (A) complete and precise diagnosis; (B) appropriate data integration within electronic health record systems and efficient machine learning to analyze big data; and (C) design, check, and deliver multimodal interventions [73]. In the last few years, the technology of digital twins (DTs) has become a field of interest [74]. It’s an interesting tool for phenotyping MS patients by creating a virtual copy (twin), through the analysis of a large quantity of data (big data) [74]. The DTs provide individual therapy simulation in advance and visualize potential therapy results and disease progression [74]. However, the use of DTs in medical care is in its early stage, only time could answer the question of whether DTs will be a game changer in the management of MS [74].

Innovative therapeutic target in MS is an active field. Besides the development of new DMTs and MLMs, AI helps design the next generation of nanorobotics [75]. These nanorobotics will go through the blood-brain barrier and make a direct therapeutic delivery to the CNS inflammatory lesions [75]. This emerging application is in constant development, to have more efficient theranostic nanobots for MS in the future [75]. Another implication of AI in the management of MS is rehabilitation technology [76]. In recent years, robot-assisted gait training (RAGT) has been proposed as a therapy for balance and gait dysfunctions in people with MS [77,78]. It has shown its effectiveness in recovering gait speed and resistance which reduces disabilities of people with MS [79].

## Conclusions

AI has the potential to revolutionize personalized medicine approaches in MS. By integrating large-scale patient data, including genomic information, biomarkers, imaging data, and real-world evidence, AI models can provide more precise and tailored treatment recommendations based on individual patient characteristics. AI algorithms can leverage longitudinal patient data and real-time monitoring to predict disease progression, relapse risk, and treatment response. Even with the advantages of AI in MS management, a huge gap exists in the use and development of AI between developed and developing countries. This gap is due to data shortage, limited resources, skills gap, and infrastructure constraints. To reduce the AI gap, developing countries can prioritize AI education at all levels, improve technological infrastructure, share data resources, and promote international collaborations.

Although AI brings numerous benefits to MS care, it also raises ethical considerations. Ensuring data privacy, maintaining transparency in AI algorithms, and addressing biases are crucial aspects to be addressed. Additionally, the integration of AI into clinical practice requires appropriate training, validation, and collaboration between healthcare professionals and AI experts. The future of AI in MS involves collaborative learning and knowledge sharing among researchers, clinicians, and patients from all countries. By sharing data, algorithms, and insights across institutions and countries, AI models can be trained on diverse and representative datasets, leading to more robust and generalizable models that benefit the entire MS community.
